# Outcomes in Atrial Fibrillation Patients with Different Clinical Phenotypes: Insights from the French Population

**DOI:** 10.3390/jcm14041044

**Published:** 2025-02-07

**Authors:** Ameenathul M. Fawzy, Arnaud Bisson, Lisa Lochon, Thibault Lenormand, Gregory Y. H. Lip, Laurent Fauchier

**Affiliations:** 1 Liverpool Centre for Cardiovascular Science at University of Liverpool, Liverpool John Moores University and Liverpool Heart & Chest Hospital, Liverpool L14 3PE, UK; ameenathul.fawzy@nhs.net (A.M.F.); lipgy@liverpool.ac.uk (G.Y.H.L.); 2 Cardiology Department, Tours Regional University Hospital and Medical School, University of Tours, Avenue de la République, 37044 Tours, France; arnaud.bisson@univ-tours.fr (A.B.); lisa.lochon@outlook.com (L.L.); lenormand.thibault@gmail.com (T.L.); 3 Cardiology Department, Orleans University Hospital, 45067 Orléans, France

**Keywords:** atrial fibrillation, clusters, phenotypes, risk, adverse outcomes

## Abstract

**Background**: Atrial fibrillation (AF) patients represent a clinically complex, heterogeneous population comprising multiple homogeneous cohorts. **Purpose:** We aimed to identify the common clinical phenotypes of AF patients and compare clinical outcomes between these subgroups. **Methods:** A 1% representative sample of all AF patients hospitalized between 2010 and 2019 was identified from the French national database. Agglomerative hierarchical cluster analysis was performed using Ward’s method and squared Euclidian distance to derive the clusters of patients. Cox regression analyses were used to evaluate outcomes including all-cause death, cardiovascular death, non-cardiovascular death, ischemic stroke, hospitalization for heart failure (HF) and composite of ventricular tachycardia, ventricular fibrillation and cardiac arrest (VT/VF/CA) over a mean follow-up period of 2.0 ± 2.3 years. **Results:** Four clusters were generated from the 12,688 patients included. Cluster 1 (*n* = 2375) was younger, low cardiovascular disease (CVD)-risk group with a high cancer prevalence. Clusters 2 (*n* = 6441) and 3 (*n* = 1639) depicted moderate-risk groups for CVD. Cluster 3 also had the highest degree of frailty and lung disease while Cluster 4 (*n* = 2233) represented a high-risk cohort for CVD. After adjusting for confounders, with cluster 1 as the reference, cluster 3 had the highest risk of all-cause death, HR 1.24 (1.09–1.41), ARD (10.3%), cardiovascular death, HR 1.56 (1.19–2.06), ARD (3.3%), non-cardiovascular death, HR 1.20 (1.04–1.38), ARD (6.9%), hospitalization for HF, HR 2.07 (1.71–2.50), ARD (9.1%) and VT/VF/CA, HR 1.74 (1.20–2.53), (ARD 1.3%). **Conclusions:** Four distinct clusters of AF patients were identified, discriminated by the differential presence of comorbidities. Our findings suggest that hospitalized AF patients with moderate CVD risk may have a poorer prognosis compared to hospitalized AF patients with high CVD risk in the presence of lung pathology and frailty. This subgroup of patients may require more stringent management of existing comorbidities such as chronic obstructive pulmonary disease and sleep apnea, alongside their AF.

## 1. Introduction

Atrial fibrillation (AF) patients represent a population that is heterogeneous and clinically complex, owing to the myriad of co-morbidities and varying degrees of disease severity. This in turn can translate into risk, influencing short- and long-term prognosis. Although recent approaches to AF management emphasize treatment of cardiovascular risk factors and concomitant diseases, and advocate for holistic care, they are broadly generalized and limited in their ability to distinguish between subsets of patients that are more likely to experience poorer outcomes and therefore require closer attention [1,2]. It is of utmost significance to recognize differences between these AF subgroups instead of viewing them as a single entity, to facilitate patient-centered care. After all, risk in AF is incongruous and cannot be assumed uniform.

Conventional studies have utilized a diverse range of statistical methods to identify markers associated with poor outcomes, but these have primarily been based on pre-specified study groups. Cluster analyses, in contrast, are exploratory and conducted such that the subgroups are determined after the application of statistical methodologies, meaning that they are not pre-meditated. Hence, they offer the opportunity to make new observations that may not be apparent and identify homogeneous cohorts of patients that comprise the larger, heterogeneous AF population. This can enable appreciation of the similarities within clusters as well as the dissimilarities between clusters of patients, allow for comparisons to be made between them and facilitate the identification of at-risk cohorts. This is endorsed in the recent European Society of Cardiology (ESC) guidelines, which emphasize delivering patient-centered, multidisciplinary care through the AF-CARE pathway. A key component of this pathway is comorbidity and risk factor management, which takes into consideration the unique circumstances and risk profile of each patient [1].

To date, few cluster analyses have been performed [3,4,5], primarily on patients from randomized controlled trials (RCTs) and registry studies aiding characterization of the clinical phenotypes in these cohorts, but data from real-world populations are lacking. Hence, we performed a cluster analysis in a real-world population of French patients with AF.

## 2. Methods

### 2.1. Study Design and Population

This retrospective, observational study was conducted across the French population. Study participants were identified from the National Hospital database, *Programme de Médicalisation des Systèmes d’Information* (PMSI), an administrative database that holds medical information on every individual admitted to hospital across the entire nation from birth or immigration to death or emigration, accounting for over 98% of the French population. The system includes data relevant to all hospitalizations such as patient demographics, clinical presentations, diagnoses and procedures. Patient details are anonymized and recorded at the time of admission, and diagnoses are encoded at discharge according to the International Classification of Diseases, Tenth Revision (ICD-10). The Classification Commune des Actes Médicaux (CCAM) was used to retrieve information related to procedures. This database has been widely used to study patients with cardiovascular conditions including those with AF [6,7,8,9,10].

AF patients hospitalized in French hospitals between 2010 and 2019 were identified from PMSI. From this, a 1% representative sample was selected. The dataset from this sample used did not contain any missing data. As the study was retrospective and conducted using anonymized data without direct patient involvement, ethical approval was not required. Access to the PMSI data was granted by the French Data Protection Authority, and procedures for handling the data were approved by the independent national ethics committee. All study proceedings were carried out according to the Declaration of Helsinki.

### 2.2. Follow-Up and Outcomes

Baseline was the date of first admission to hospital, and follow-up was continued until 31 December 2019 or until death/emigration. The mean follow-up duration was 2.0 ± 2.3 years (median 1.1 years, [interquartile range 0.1–3.4]).

Outcomes evaluated were all-cause death, cardiovascular death, non-cardiovascular death, ischemic stroke, hospitalization for heart failure (HF) and the composite outcome of ventricular tachycardia (VT), ventricular fibrillation (VF) and cardiac arrest (CA) during the follow-up period. Diagnoses and outcomes were identified using respective ICD-10 codes (Supplementary Table S1) as adjudicated by physicians during hospitalization. The mode of death (cardiovascular or non-cardiovascular) was identified based on the main diagnosis during hospitalization resulting in death (ICD-codes: cardiovascular death: I00–I99).

Patients were excluded from the analysis if the outcome event was experienced during the same admission as the AF diagnosis.

### 2.3. Statistical Analysis

Continuous variables were expressed as mean (±standard deviation, SD), and categorical variables were expressed as counts and percentages. Differences between groups were assessed using *t*-tests for continuous variables and the chi-squared χ^2^ test for categorical variables.

A 1% representative sample of AF patients was used for the clustering process. Agglomerative hierarchical clustering was chosen for this analysis as it is particularly well-suited for exploratory studies like this one, where the number of clusters need not be predefined and instead be determined using a dendrogram. Additionally, this method offers greater flexibility as it performs well with complex, irregularly shaped clusters of varying sizes and is less impacted by the effects of outliers as opposed to some of the other clustering methods such as K-means. A comparison of the common clustering models that were taken into account is provided in Supplementary Table S2. Prior papers have also used relatively similar methods for AF [3,5].

Agglomerative hierarchical clustering was performed using Ward’s method and squared Euclidian distance, using the following 31 clinical variables: age, sex, hypertension, diabetes, heart failure, history of pulmonary oedema, aortic regurgitation, mitral regurgitation, previous endocarditis, dilated cardiomyopathy, coronary artery disease, previous myocardial infarction, previous percutaneous coronary intervention (PCI), coronary artery bypass graft (CABG) surgery, vascular disease, pacemaker or implantable cardioverter-defibrillator (ICD), ischemic stroke, intracranial bleeding, smoking status, dyslipidemia, obesity, alcohol-related disorders, abnormal renal function, lung disease, sleep apnea, chronic obstructive pulmonary disease (COPD), liver disease, thyroid disease, inflammatory disease, anemia and previous cancer. Multicollinearity between the variables was assessed using variation inflation factor (VIF). None of the variables had a VIF score of >5 to indicate high multicollinearity. The highest VIF score was observed for hypertension (VIF = 2.19), followed by lung disease (VIF = 2.17), and the mean VIF was 1.23. Therefore, no variables were removed, and all 31 were included in the analysis.

**Figure 1:**
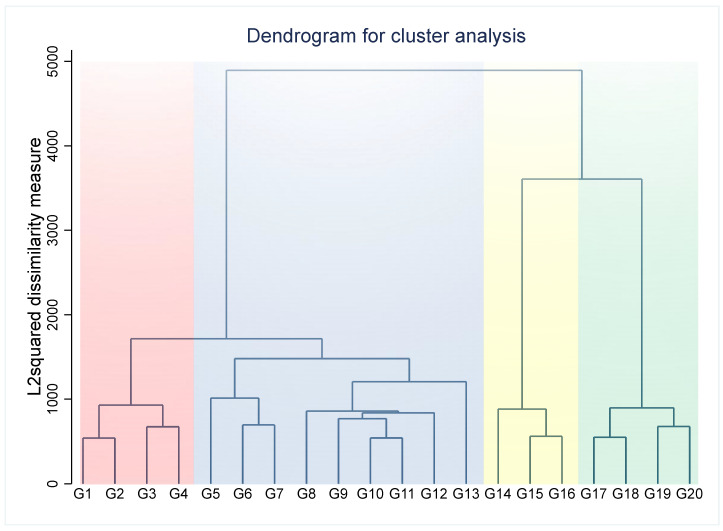
Dendrogram generated by hierarchical clustering process showing the 4 AF clusters. The dendrogram graph is the visual representation of the hierarchical clustering process. Vertical lines are clusters that are joined together, and the position of the line on the scale indicates the distance at which clusters were joined.

Agglomerative hierarchical clustering is based on a ‘bottom-to-top’ approach where the clustering begins with a single patient, who is then grouped with another based on similarities in the specified clinical variables. In this way, small patient clusters are formed and merged to form bigger clusters that ultimately make up the entire study population. The dendrogram (Figure 1) provides a visual depiction of the clustering process with the vertical lines representing the various clusters and the distance between the clusters equating to the sum of squared differences within all the clusters. This was used to determine the number of clusters.

Outcome events were then evaluated between the four clusters. Incidence rates were expressed in percentage (%)/year. Risk of outcome events between clusters were evaluated using Cox regression models and expressed as hazard ratios (HRs). Univariate and multivariate analyses were performed with Model 1 adjusted for age and sex and model 2 adjusted for the CHA_2_DS_2_VASc (1 point each for C—congestive cardiac failure, H—hypertension, age—65–74 years, V—vascular disease and S—female sex and 2 points each for A—age ≥ 75 years and S—stroke) score. Absolute risk differences (ARD) werepresented as the difference in the cumulative incidence of each outcome between clusters. All tests were 2-tailed, and *p*-values of ≤0.05 were considered statistically significant.

Statistical analysis was performed using Enterprise Guide 7.1, (SAS Institute Inc., Cary, NC, USA) and STATA version 16.0 (Stata Corp, College Station, TX, USA). Results were presented according to the Strengthening the Reporting of Observational Studies in Epidemiology (STROBE) reporting guidelines (Supplementary Table S3).

## 3. Results

**Figure 2:**
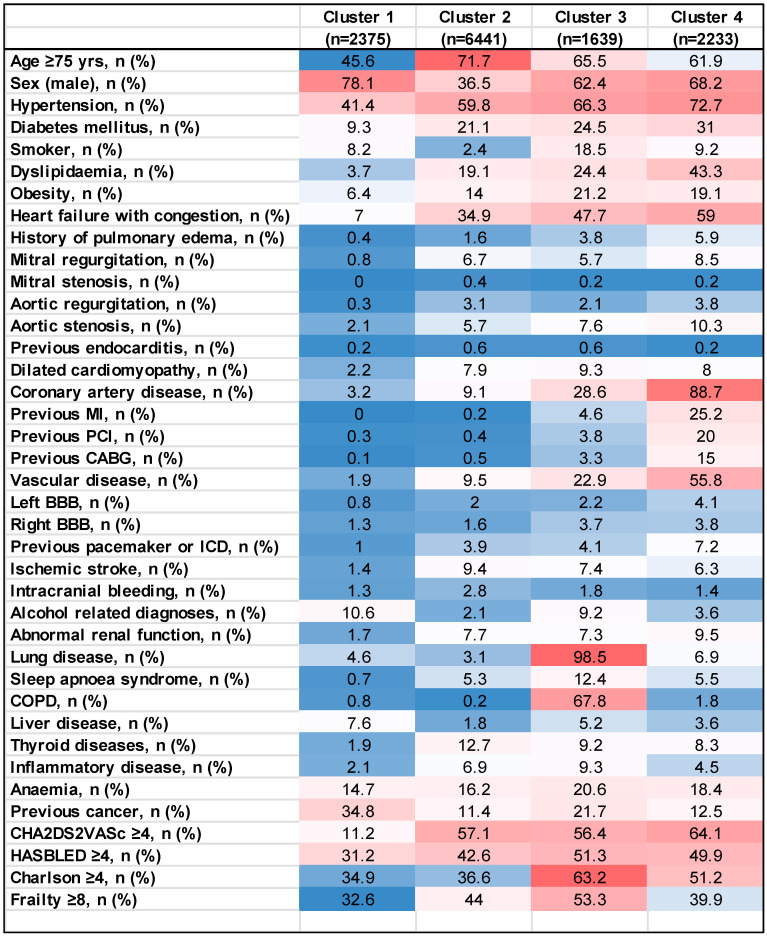
Heatmap of the baseline characteristics of AF patients according to the patient clusters. Legend for heat map colors: from blue (low %: represents the lowest values in the dataset) to red (highest %: highlights the maximum values in the dataset).

A total of 12,688 patients were included, and four clusters were identified. Each of these had distinct characteristics that distinguished one from the other (Table 1). A heatmap of the baseline characteristics of AF patients according to the patient clusters is shown in Figure 2.

### 3.1. Baseline Characteristics

#### 3.1.1. Cluster 1 (*n* = 2375)

Cluster 1 had the youngest patients with a mean age of 71.6 ± 13.7 years and mostly comprised of males (78.1%). This group had the lowest prevalence of cardiovascular risk factors and comorbidities such as hypertension, diabetes, dyslipidemia, smoking, coronary artery disease, vascular disease and HF. The prevalence of respiratory conditions including sleep apnea and chronic obstructive pulmonary disease (COPD) was also minimal in this cluster. Stroke and bleeding risks were lowest in this group, with mean CHA_2_DS_2_VASc and HASBLED scores of 2.0 ± 1.3 and 2.0 ± 1.3, respectively. Thus, cluster 1 represented a low-risk population, which was fairly robust, evidenced by their relatively low Charlson and frailty indices. However, this group had the highest proportion of patients with a history of cancer.

#### 3.1.2. Cluster 2 (*n* = 6441)

Cluster 2 was the largest subset, the oldest, with a mean age of 78.9 ± 11.7 years and the only cluster with a female predominance (63.5%). This group had the highest proportion of patients with prior ischemic stroke and intracranial bleeding and the lowest prevalence of smoking and lung disease. The mean CHA_2_DS_2_VASc and HASBLED scores were 3.7 ± 1.4 and 2.3 ± 1.1, respectively, indicative of a high stroke and intermediate bleeding risk. The patients were more frail compared to clusters 1 and 4. Overall, this cluster represented a moderate–severe frail cohort with a moderate cardiovascular risk and high stroke risk.

#### 3.1.3. Cluster 3 (*n* = 1639)

Cluster 3 was the smallest with a mean age of 77.9 ± 10.4 years. Male sex accounted for 62.4% of the cohort. This group was characterized by the highest prevalence of smokers (18.5%) and lung disease, which was present in 98.5% of patients. Of these, 67.8% of patients had COPD and 12.4% of patients had sleep apnea. Over half the patients had a history of hypertension, nearly half had HF, and a quarter had other risk factors such as diabetes and dyslipidemia. Patients in this group had high stroke and bleeding risks with a mean CHA_2_DS_2_VASc score of 3.7 ± 1.6 and HASBLED score of 2.6 ± 1.1. This group also had the highest level of frailty, indicated by mean Charlson and frailty indices of 4.7 ± 2.7 and 10.7 ± 8.6, respectively. Hence, cluster 3 represented a severely frail cohort with moderate cardiovascular risk and high prevalence of lung pathologies.

#### 3.1.4. Cluster 4 (*n* = 2233)

The mean age of cluster 4 was 76.9 ± 10.7 years, and 68.2% of this group was male. Cluster 4 was signified by the highest prevalence of cardiovascular risk factors and comorbidities such as hypertension, diabetes, HF and valvulopathies. A total of 88.7% of patients in this cohort had established coronary artery disease, and 55.8% had vascular disease. Compared to the other clusters, it also had the greatest proportion of individuals with kidney disease, procedures such as PCI and CABG surgery as well as pacemakers and ICDs. Consequently, this group had the highest mean CHA_2_DS_2_VASc score (4.1 ± 1.7), but the HAS-BLED score was comparable to that of cluster 3 (2.6 ± 1.2). Patients were moderately frail relative to the other clusters with Charlson and frailty indices of 4.1 ± 3.0 and 8.7 ± 8.9, respectively.

### 3.2. Major Adverse Cardiovascular Events

**Figure 3:**
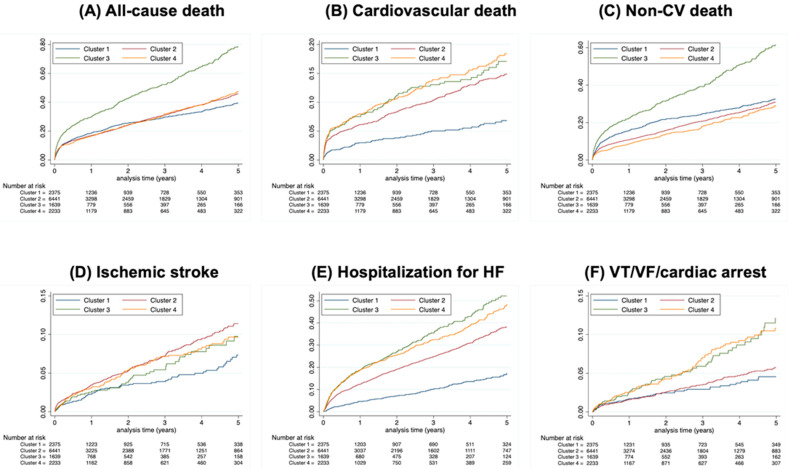
**Cumulative incidences for study outcomes.** Cumulative incidences for all-cause death (**A**), cardiovascular death (**B**), non-cardiovascular death (**C**), ischemic stroke (**D**), rehospitalization for HF (**E**) or VT/VF/CA (**F**) during follow-up in patients with AF according to patient clusters.

A total of 9135 events were observed over a follow-up period of 2.0 ± 2.3 years. Of these, 1354, 4446, 1617 and 1718 events were observed in clusters 1, 2, 3 and 4, respectively. Incidence rates of these events are summarized in Table 2, and cumulative incidences are demonstrated in Figure 3. ARDs for each outcome are presented in Table 2. Risk was evaluated using cluster 1 as a reference (Table 3).

#### 3.2.1. All-Cause Death

The incidence of all-cause death progressively increased from cluster 1 to cluster 4, then cluster 2 and finally cluster 3, where the incidence rate was twice as high as cluster 1 (20.7%/years versus 10.4%/year). After adjusting for age and sex, cluster 2 had a lower risk of death compared to cluster 1, HR 0.88 (0.79–0.98), which remained statistically significant, in model 2, HR 0.71 (0.64–0.79). Cluster 3 had a significantly higher risk in model 1, HR 1.55 (1.37–1.74), which was attenuated in model 2, HR 1.24 (1.09–1.41). In contrast, there was no difference in the risk of all-cause death between clusters 1 and 4 in model 1, HR 0.93 (0.82–1.05), but a significantly lower risk of mortality was observed in model 2, HR 0.64 (0.56–0.73) for cluster 4.

#### 3.2.2. Cardiovascular Death

Incidence of cardiovascular death progressively increased from cluster 1 to clusters 2, 4 and 3. Compared to cluster 1, the risk of cardiovascular death was significantly higher in cluster 2 in model 1, HR 1.70 (1.34–2.15), but no longer significant in model 2, HR 1.24 (0.98–1.58). For cluster 3, the risk was significantly higher in models 1 and 2 with HR 2.17 (1.66–2.83) and HR 1.56 (1.19–2.06), respectively. A similar pattern was observed for cluster 4, where HR was 2.26 (1.76–2.90) in model 1 and HR 1.34 (1.03–1.75) in model 2.

#### 3.2.3. Non-Cardiovascular Death

The risk of non-cardiovascular death was lower in clusters 2 and 4 compared to cluster 1. This may have been driven by the higher prevalence of neoplasms in cluster 1. The risk for cluster 2 was HR 0.71 (0.63–0.81) and HR 0.60 (0.53–0.68) in models 1 and 2, respectively, whereas the corresponding risks for cluster 4 were HR 0.65 (0.56–0.76) and HR 0.48 (0.41–0.57), respectively. The non-cardiovascular mortality risk was significantly higher in cluster 3 and was HR 1.43 (1.25–1.63) in model 1 and HR 1.20 (1.04–1.38) in model 2. This may be attributable to the higher prevalence of non-cardiovascular co-morbidities such as lung pathologies—further evidenced by the high frailty index.

#### 3.2.4. Ischemic Stroke

For ischemic stroke, only cluster 2 had a significantly elevated risk compared to cluster 1, HR 1.41 (1.09–1.83) in model 1. However, this was no longer significant after adjusting for the CHA_2_DS_2_VASc score (model 2), HR 0.99 (0.76–1.29). No significant differences in risk were observed for clusters 3 and 4.

#### 3.2.5. Hospitalization for HF

HF hospitalization risk was significantly elevated in all clusters compared to cluster 1. For cluster 2, the risk was HR 0.99 (0.76–1.29) in model 1 and HR 1.48 (1.26–1.75) in model 2, and for cluster 3, the risk was HR 2.69 (2.23–3.24) and HR 2.07 (1.71–2.50) in models 1 and 2, respectively. Corresponding risks were HR 2.68 (2.26–3.19) and HR 1.76 (1.46–2.11), respectively, for cluster 4. Despite the higher proportion of patients with HF and previous pulmonary oedema, cluster 4 had a lower risk of hospitalization for heart failure relative to cluster 3.

#### 3.2.6. VT/VF/CA

There were no significant differences in the risk of VT/VF/CA between clusters 1 and 2. However, this risk was significantly higher in cluster 3 in model 1, HR 2.16 (1.50–3.09), and model 2, HR 1.74 (1.20–2.53); and cluster 4 in model 1, HR 1.98 (1.42–2.76), and model 2, HR 1.56 (1.09–2.23), compared to cluster 1.

## 4. Discussion

To our knowledge, this is one of the first cluster analyses to be performed on a real-world cohort of patients with AF. We identified four distinct AF phenotypes—younger, healthier patients, with low stroke, bleeding, and cardiovascular risks and mild frailty (cluster 1); older patients with intermediate stroke, bleeding, and cardiovascular risks and moderate frailty (cluster 2); older patients with high stroke and bleeding risks, high burden of lung disease and severe frailty (cluster 3); and older patients with high stroke and bleeding risks, moderate cardiovascular risk and moderate–severe frailty (cluster 4). Our findings emphasize the heterogeneity of the AF population and, importantly, indicate that risk between subgroups of AF patients varies depending on existing co-morbidities and clinical characteristics. Even though AF patients with cardiovascular comorbidities will have been predicted to have the poorest prognosis, we observed that this was not wholly the case.

In our study, cluster 3, characterized by a high burden of lung disease, present in nearly all patients, and severe frailty, demonstrated the highest risk for all outcomes except ischemic stroke, where no significant differences were observed between the groups. This was despite cluster 4, which was predominated by cardiovascular risk factors and comorbidities such as coronary artery disease, HF and vascular disease. Nonetheless, the risk of outcomes, even those that were cardiovascular-specific, was higher in cluster 3 compared to cluster 4, suggesting that AF patients with moderate CVD risk have a poorer prognosis compared to those with high CVD risk, in the presence of lung disease and advancing frailty. Frailty is increasingly being recognized as a marker of poor prognosis, increasing the risk of adverse outcomes such as ischemic stroke, bleeding and mortality in AF patients. In the meta-analysis by Proietti et al., the odds of all-cause death were nearly six times more in frail individuals with AF compared to those who were more robust [11].

The association between AF and respiratory disease has been less well-studied and only recognized in recent years, with emerging evidence demonstrating poor outcomes in the presence of respiratory conditions such as COPD and obstructive sleep apnea (OSA) [12,13]. Recent studies suggest that these conditions may increase the risk of AF through mechanisms such as hypercapnia and hypoxemia, which can produce pulmonary arteriolar constriction and result in pulmonary and right ventricular hypertension. This, in turn, can result in increased right ventricular overload and right atrial dilatation, amongst other changes, increasing susceptibility to AF [14,15,16]. Chronic lung disease has also been linked to a higher prevalence of atypical foci for AF genesis, which are associated with higher failure rates of treatments such as catheter ablation and poorer outcomes [17,18]. Recently, a RCT demonstrated that continuous positive airway pressure therapy in AF patients with OSA led to reversal of atrial remodeling, advocating for management of these comorbidities as a crucial component to mitigating risk in AF [19].

To date, few cluster analyses have been performed using data from registry studies, highlighting the common AF phenotypes found in every population, but also identifying unique clusters of AF patients present only in certain populations. In the cluster analysis by Inohara et al., an atherosclerotic cluster was identified from the ORBIT registry, similar to cluster 4 in our study, but two distinct clusters were also identified; the younger-behavioral and device-dependent clusters had a higher risk of outcomes compared to the younger-healthy cluster [4]. Analysis from the EORP-AF registry identified three distinct clusters; a young, healthy cluster, older cluster with cardiovascular co-morbidities and older cluster with non-cardiovascular comorbidities. The latter two cohorts had poorer outcomes with the risk being higher in the cluster with cardiovascular co-morbidities. That said, this cluster also had the greatest proportion of patients with frailty though proportions of patients with COPD and OSA were similar between the two [3].

In the analysis by Vitolo et al., which used data from the AMADEUS and BOREALIS RCTs, the cluster with the highest rates of non-cardiovascular comorbidities had the poorest outcomes, though a considerable proportion of patients also had pre-existing cardiovascular risk factors. Additionally, there were two separate clusters, one with high cardiovascular comorbidities but low burden of cardiovascular events and the other with a high burden of both cardiovascular risk factors and comorbidities. However, no significant differences were observed in the composite outcome, all-cause death and major bleeding in these clusters compared to the lower risk cluster [5].

In our study, cluster 1 was deemed the lowest risk cluster from a cardiovascular point of view. However, there was a high prevalence of cancer, which may explain the high risk of all-cause death and non-cardiovascular death compared to clusters 2 and 4. Cluster 3, however, demonstrated a significantly higher risk for these outcomes as well as cardiovascular death. For the remaining outcomes of interest, all other clusters demonstrated a higher risk compared to cluster 1.

Although the clusters generated can vary depending on the population being studied and direct comparisons of risk between clusters cannot be made due to differences in the variables considered, the strength of the cluster analysis lies in its ability to singularize distinct phenotypic subgroups that may or may not be driving outcomes. Regarding the latter, cluster analyses performed by Pastori et al. [20]. and Inohara et al. [21]. demonstrated that factors such as AF type and left atrial size did not significantly influence outcomes events, despite driving cluster formations.

In contrast, in cluster analyses performed thus far, we commonly observed that the highest risk of adverse events was not in AF patients with cardiovascular risk factors and morbidities alone, but in those with other non-cardiovascular comorbidities. This may partly be reflective of the recent leaps made in the treatment of various cardiovascular and metabolic conditions, especially with the increased uptake of drugs such as sodium glucose co-transporter 2 (SGLT2) inhibitors and glucagon-like peptide-1 (GLP-1) agonists that have demonstrated significant benefits in terms of reducing morbidity and mortality, both in RCTs and observational studies [22,23,24,25,26,27,28,29,30,31,32,33,34,35]. The impact of these drugs has also been examined specifically in AF populations, with some, particularly studies of SGLT2 inhibitors, indicating reduced risks of outcomes such as AF-related hospitalizations, stroke and ventricular arrhythmias in addition to death and HF hospitalizations [36,37,38,39,40]. Unfortunately, the influence of these drugs on our results could not be evaluated as data on medications was unavailable.

The number of comorbidities also appeared to make a difference, indicative of the frailty status as per the cumulative deficit model [41]. Ischemic stroke rates between these clusters of AF patients were no different, but the stark variations in some of the other outcomes highlight that long-term management of AF patients extends beyond anti-coagulation and emphasizes the importance of adhering to the guideline recommended ‘ABC’ or ‘CARE-AF’ pathways. The ‘C’ component of this integrated approach signifies detection and management of cardiovascular risk factors and concomitant diseases, which explicitly includes OSA, largely as a mean to reduce AF burden, recurrence and symptom severity. Our findings advocate for the diagnosis and management of such comorbidities beyond these purposes, to reduce the risk of adverse outcomes such as mortality, HF and ventricular arrhythmias. Further, the phenotypic clusters that highlight the distinct comorbidities and risk profiles within the AF population offer a valuable basis for personalized, patient-centered management of AF.

As cluster analyses are purely exploratory, further research needs to be carried out to confirm our findings, understand the complex interplay between various comorbidities that may be driving outcomes, elucidate if adequate management of certain conditions can minimize adverse outcomes and determine the extent to which this risk can be reduced.

### Limitations

As this method of clustering cannot be performed on very large datasets, a 1% sample representative of the AF population was used for the purpose of the study. That said, the sample picked was from a hospitalized AF population that may exhibit more severe disease phenotypes, comorbidities and treatment pathways and may not be representative of the wider AF population, inclusive of those managed in the community, whose characteristics may be different. Further, it may not have captured rare but clinically relevant phenotypes. Hence, there may be elements of sampling bias potentially influencing results, and a different set of clusters may have been derived depending on the patient population and clustering method selected. Despite this limitation, the robustness of our findings is supported by the use of agglomerative hierarchical clustering, the strength of which lies in its stability with smaller sample sizes and ability to reveal hierarchical relationships within the dataset. After all, the key utility of this method of analysis is to identify the smaller, clinically relevant and often less apparent subgroups that exist within the broader AF population. Nonetheless, the results of this study may not be generalizable, and further studies are required to validate these findings in larger, more representative populations to assess generalizability. It must also be emphasized that although clusters with similar characteristics that demonstrate similar trends in outcomes can be hypothesis generating, direct comparisons regarding risk cannot be made between clusters across different populations and causal inferences cannot be drawn.

Further, data on medications such as anti-arrhythmic drugs used directly in the treatment of AF, as well as those such as sodium glucose co-transporter 2 inhibitors and glucagon-like peptide 1 receptor agonists that may have wider benefits in terms reducing cardiovascular events and AF-related complications, were not available [22,42,43]. This, as well as unmeasured confounders, may have contributed to the results and limits attributability of the observed outcomes purely to the phenotypes derived. The analysis was retrospective, using administrative data, and although significant efforts are made to ensure comprehensive and correct coding, potential coding inaccuracies may still exist, introducing a degree of information bias. The study is also subject to bias due to time-varying confounding and competing risks. Additionally, external validation of the analysis was not possible due to lack of access to an independent database with the same variables. Lastly, interaction analyses were not performed, and it was unclear whether there were certain factors within clusters (e.g., lung pathologies or frailty in cluster 3) that were more likely to contribute to the outcomes.

## 5. Conclusions

In this analysis of French AF patients, we identified four distinct phenotypic clusters, characterized by the differential presence of comorbidities and unique risk profiles. Hospitalized AF patients with moderate CVD risk, high prevalence of respiratory conditions and greater degree of frailty had a poorer prognosis compared to hospitalized AF patients with high CVD risk but lower burden of lung pathologies. Therefore, this subgroup of patients requires further scrutinization and more stringent management of existing comorbidities such as COPD and sleep apnea, alongside their AF to minimize risk of adverse outcomes.

## Figures and Tables

**Table 1:** Baseline characteristics of patients with AF seen in French hospitals (2010–2019) according to patient clusters.

	Cluster 1	Cluster 2	Cluster 3	Cluster 4	*p*	Total
	(*n* = 2375)	(*n* = 6441)	(*n* = 1639)	(*n* = 2233)		(*n* = 12,688)
Age (years), mean ± SD	71.6 ± 13.7	78.9 ± 11.7	77.9 ± 10.4	76.9 ± 10.7	<0.0001	77.1 ± 12.1
Sex (male), *n* (%)	1855 (78.1)	2353 (36.5)	1022 (62.4)	1523 (68.2)	<0.0001	6753 (53.2)
Hypertension, *n* (%)	984 (41.4)	3850 (59.8)	1086 (66.3)	1623 (72.7)	<0.0001	7543 (59.5)
Diabetes mellitus, *n* (%)	220 (9.3)	1360 (21.1)	401 (24.5)	692 (31.0)	<0.0001	2673 (21.1)
Smoker, *n* (%)	195 (8.2)	155 (2.4)	303 (18.5)	206 (9.2)	<0.0001	859 (6.8)
Dyslipidemia, *n* (%)	87 (3.7)	1233 (19.1)	400 (24.4)	966 (43.3)	<0.0001	2686 (21.2)
Obesity, *n* (%)	151 (6.4)	903 (14.0)	348 (21.2)	426 (19.1)	<0.0001	1828 (14.4)
Heart failure with congestion, *n* (%)	167 (7.0)	2247 (34.9)	781 (47.7)	1317 (59.0)	<0.0001	4512 (35.6)
History of pulmonary edema, *n* (%)	10 (0.4)	101 (1.6)	62 (3.8)	132 (5.9)	<0.0001	305 (2.4)
Mitral regurgitation, *n* (%)	18 (0.8)	434 (6.7)	93 (5.7)	189 (8.5)	<0.0001	734 (5.8)
Mitral stenosis, *n* (%)	1 (0.0)	23 (0.4)	3 (0.2)	4 (0.2)	0.02	31 (0.2)
Aortic regurgitation, *n* (%)	8 (0.3)	197 (3.1)	34 (2.1)	84 (3.8)	<0.0001	323 (2.5)
Aortic stenosis, *n* (%)	49 (2.1)	368 (5.7)	125 (7.6)	231 (10.3)	<0.0001	773 (6.1)
Previous endocarditis, *n* (%)	4 (0.2)	37 (0.6)	10 (0.6)	4 (0.2)	0.003	55 (0.4)
Dilated cardiomyopathy, *n* (%)	53 (2.2)	512 (7.9)	153 (9.3)	178 (8.0)	<0.0001	896 (7.1)
Coronary artery disease, *n* (%)	75 (3.2)	583 (9.1)	468 (28.6)	1981 (88.7)	<0.0001	3107 (24.5)
Previous MI, *n* (%)	0 (0.0)	12 (0.2)	76 (4.6)	563 (25.2)	<0.0001	651 (5.1)
Previous PCI, *n* (%)	7 (0.3)	25 (0.4)	63 (3.8)	447 (20.0)	<0.0001	542 (4.3)
Previous CABG, *n* (%)	3 (0.1)	30 (0.5)	54 (3.3)	334 (15.0)	<0.0001	421 (3.3)
Vascular disease, *n* (%)	46 (1.9)	611 (9.5)	376 (22.9)	1245 (55.8)	<0.0001	2278 (18.0)
Left BBB, *n* (%)	20 (0.8)	126 (2.0)	36 (2.2)	91 (4.1)	<0.0001	273 (2.2)
Right BBB, *n* (%)	31 (1.3)	105 (1.6)	60 (3.7)	84 (3.8)	<0.0001	280 (2.2)
Previous pacemaker or ICD, *n* (%)	24 (1.0)	250 (3.9)	68 (4.1)	160 (7.2)	<0.0001	502 (4.0)
Ischemic stroke, *n* (%)	34 (1.4)	603 (9.4)	121 (7.4)	140 (6.3)	<0.0001	898 (7.1)
Intracranial bleeding, *n* (%)	30 (1.3)	181 (2.8)	30 (1.8)	31 (1.4)	<0.0001	272 (2.1)
Alcohol related diagnoses, *n* (%)	252 (10.6)	138 (2.1)	151 (9.2)	80 (3.6)	<0.0001	621 (4.9)
Abnormal renal function, *n* (%)	40 (1.7)	493 (7.7)	120 (7.3)	212 (9.5)	<0.0001	865 (6.8)
Lung disease, *n* (%)	109 (4.6)	198 (3.1)	1614 (98.5)	154 (6.9)	<0.0001	2075 (16.4)
Sleep apnea syndrome, *n* (%)	16 (0.7)	339 (5.3)	203 (12.4)	123 (5.5)	<0.0001	681 (5.4)
COPD, *n* (%)	20 (0.8)	16 (0.2)	1112 (67.8)	40 (1.8)	<0.0001	1188 (9.4)
Liver disease, *n* (%)	181 (7.6)	117 (1.8)	85 (5.2)	81 (3.6)	<0.0001	464 (3.7)
Thyroid diseases, *n* (%)	44 (1.9)	815 (12.7)	150 (9.2)	185 (8.3)	<0.0001	1194 (9.4)
Inflammatory disease, *n* (%)	49 (2.1)	444 (6.9)	153 (9.3)	100 (4.5)	<0.0001	746 (5.9)
Anemia, *n* (%)	350 (14.7)	1041 (16.2)	338 (20.6)	410 (18.4)	<0.0001	2139 (16.9)
Previous cancer, *n* (%)	826 (34.8)	737 (11.4)	356 (21.7)	279 (12.5)	<0.0001	2198 (17.3)
CHA2DS2VASc score, mean ±SD	2.0 ± 1.3	3.7 ± 1.4	3.7 ± 1.6	4.1 ± 1.7	<0.0001	3.4 ± 1.6
HASBLED score, mean ± SD	2.0 ± 1.3	2.3 ± 1.1	2.6 ± 1.1	2.6 ± 1.2	<0.0001	2.4 ± 1.2
Charlson index, mean ± SD	2.9 ± 2.9	3.0 ± 2.5	4.7 ± 2.7	4.1 ± 3.0	<0.0001	3.4 ± 2.8
Frailty index, mean ± SD	7.0 ± 8.2	9.3 ± 9.2	10.7 ± 8.6	8.7 ± 8.9	<0.0001	9.0 ± 9.0

AF = atrial fibrillation; BBB = bundle branch block; CABG = coronary artery bypass graft; COPD = chronic obstructive pulmonary disease; ICD = implantable cardioverter defibrillator; MI = myocardial infarction; PCI = percutaneous coronary intervention.

**Table 2:** Major adverse clinical events in AF patients according to patient clusters and ARD compared to cluster 1.

	Cluster 1	ARD	Cluster 2	ARD	Cluster 3	ARD	Cluster 4	ARD
	(*n* = 2375)	%	(*n* = 6441)	%	(*n* = 1639)	%	(*n* = 2233)	%
All-cause death	520 (10.4)	Ref	1459 (11.3)	0.9	589 (20.7)	10.3	522 (11.2)	0.8
Cardiovascular death	88 (1.8)	Ref	496 (3.8)	2.0	146 (5.1)	3.3	220 (4.7)	2.9
Non-cardiovascular death	432 (8.6)	Ref	963 (7.4)	−1.2	443 (15.5)	6.9	302 (6.5)	−2.1
Ischemic stroke	79 (1.6)	Ref	329 (2.6)	1.0	58 (2.1)	0.5	105 (2.3)	0.7
Hospitalization for HF	180 (3.8)	Ref	1037 (9.0)	5.2	314 (12.9)	9.1	470 (11.9)	8.1
VT/VF/CA	55 (1.1)	Ref	162 (1.3)	0.2	67 (2.4)	1.3	99 (2.2)	1.1
Total	1354		4446		1617		1718	

Values are *n* (incidence, %/year). Abbreviations: AF—atrial fibrillation, ARD—absolute risk difference, CA—cardiac arrest, HF—heart failure, VF—ventricular fibrillation, VT—ventricular tachycardia.

**Table 3:** Cox regression analysis for main study outcomes according to patient clusters.

	Univariate		Multivariable (Model 1)		Multivariable (Model 2)	
	HR (95% CI)	*p*	HR (95% CI)	*p*	HR (95% CI)	*p*
All-cause death						
Cluster 2 (vs. Cluster 1)	1.07 (0.97–1.18)	0.19	0.88 (0.79–0.98)	0.02	0.71 (0.64–0.79)	<0.0001
Cluster 3 (vs. Cluster 1)	1.86 (1.65–2.09)	<0.0001	1.55 (1.37–1.74)	<0.0001	1.24 (1.09–1.41)	0.001
Cluster 4 (vs. Cluster 1)	1.07 (0.95–1.21)	0.26	0.93 (0.82–1.05)	0.23	0.64 (0.56–0.73)	<0.0001
Cardiovascular death						
Cluster 2 (vs. Cluster 1)	2.14 (1.70–2.68)	<0.0001	1.70 (1.34–2.15)	<0.0001	1.24 (0.98–1.58)	0.07
Cluster 3 (vs. Cluster 1)	2.67 (2.05–3.48)	<0.0001	2.17 (1.66–2.83)	<0.0001	1.56 (1.19–2.06)	0.002
Cluster 4 (vs. Cluster 1)	2.66 (2.08–3.41)	<0.0001	2.26 (1.76–2.90)	<0.0001	1.34 (1.03–1.75)	0.03
Non-cardiovascular death						
Cluster 2 (vs. Cluster 1)	0.85 (0.76–0.95)	0.006	0.71 (0.63–0.81)	<0.0001	0.60 (0.53–0.68)	<0.0001
Cluster 3 (vs. Cluster 1)	1.69 (1.48–1.93)	<0.0001	1.43 (1.25–1.63)	<0.0001	1.20 (1.04–1.38)	0.01
Cluster 4 (vs. Cluster 1)	0.75 (0.65–0.87)	<0.0001	0.65 (0.56–0.76)	<0.0001	0.48 (0.41–0.57)	<0.0001
Ischemic stroke						
Cluster 2 (vs. Cluster 1)	1.61 (1.26–2.06)	<0.0001	1.41 (1.09–1.83)	0.009	0.99 (0.76–1.29)	0.93
Cluster 3 (vs. Cluster 1)	1.25 (0.89–1.75)	0.20	1.09 (0.78–1.54)	0.61	0.78 (0.55–1.11)	0.17
Cluster 4 (vs. Cluster 1)	1.44 (1.07–1.92)	0.02	1.29 (0.96–1.73)	0.09	0.79 (0.57–1.08)	0.14
Hospitalization for HF						
Cluster 2 (vs. Cluster 1)	2.35 (2.01–2.76)	<0.0001	0.99 (0.76–1.29)	<0.0001	1.48 (1.26–1.75)	<0.0001
Cluster 3 (vs. Cluster 1)	3.24 (2.70–3.90)	<0.0001	2.69 (2.23–3.24)	<0.0001	2.07 (1.71–2.50)	<0.0001
Cluster 4 (vs. Cluster 1)	3.09 (2.60–3.67)	<0.0001	2.68 (2.26–3.19)	<0.0001	1.76 (1.46–2.11)	<0.0001
VT/VF/CA						
Cluster 2 (vs. Cluster 1)	1.14 (0.84–1.55)	0.39	1.29 (0.94–1.78)	0.11	0.94 (0.68–1.31)	0.72
Cluster 3 (vs. Cluster 1)	2.09 (1.46–2.99)	<0.0001	2.16 (1.50–3.09)	<0.0001	1.74 (1.20–2.53)	0.004
Cluster 4 (vs. Cluster 1)	1.97 (1.41–2.73)	<0.0001	1.98 (1.42–2.76)	<0.0001	1.56 (1.09–2.23)	0.02

Model 1: adjusted on age and sex. Model 2: adjusted on CHA2DS2VASc score. Abbreviations: CA—cardiac arrest, HF—heart failure, VF—ventricular fibrillation, VT—ventricular tachycardia.

## Data Availability

The data used in this study may be available from the corresponding author upon reasonable request.

## Supplementary Materials

The following supporting information can be downloaded at: https://www.mdpi.com/article/10.3390/jcm14041044/s1.

## Abbreviations

AF—atrial fibrillation, ARD—absolute risk difference, CA—cardiac arrest, CVD—cardiovascular disease, HF—heart failure, VF—ventricular fibrillation, VT—ventricular tachycardia.
