# Supplementary material for: Outcomes in Atrial Fibrillation Patients with Different Clinical Phenotypes: Insights from the French Population
Source: J Clin Med. 2025 Feb 7;14(4):1044. doi: 10.3390/jcm14041044 (PMC11856015; doi:10.3390/jcm14041044)
Supplement: Supplementary file 1 [file jcm-14-01044-s001.zip › jcm-3361691-supplementary.pdf]

SUPPLEMENTAL DATA

Outcomes in atrial fibrillation patients with different clinical phenotypes: Insights from the French population

Ameenathul M. Fawzy MB[1], Arnaud Bisson MD[2,3], Lisa Lochon MD[2], Thibault Lenormand MD[2],

Gregory Y.H. Lip MD[1], Laurent Fauchier MD PhD[2]

[Professors Lip and Fauchier are joint senior authors]

[1]Liverpool Centre for Cardiovascular Science at University of Liverpool, Liverpool John Moores University and Liverpool Heart & Chest Hospital, Liverpool, United Kingdom

[2] Tours Regional University Hospital, Hospital Trousseau, Tours, France

[3] Orleans Regional Hospital, Orleans, France

Corresponding author:

Prof Laurent Fauchier, Service de Cardiologie, Centre Hospitalier Universitaire Trousseau, Tours, France (Tel: +33247474650; fax: +33247475019)

lfau@univ-tours.fr

CONTENTS

Supplemental Table S1 – Outcomes considered in the analysis and corresponding ICD-10 outcomes used to define them

Supplemental Table S2: Comparison of some clustering methods considered for this study

Supplemental Table S3 - STROBE Statement—checklist of items that should be included in reports of observational studies.

Supplemental Table S1 – Outcomes considered in the analysis and corresponding ICD-10 outcomes used to define them

**Outcome**	**ICD-10 codes**
Cardiovascular death	I00-I99
Non-cardiovascular death	Infectious and Parasitic Diseases: A00–B99<br>Neoplasms (Cancers): C00–D48<br>Endocrine, Nutritional, and Metabolic Diseases: E00–E88<br>Mental and Behavioral Disorders: F01–F99<br>Diseases of the Nervous System: G00–G98<br>Diseases of the Respiratory System: J00–J98<br>Diseases of the Digestive System: K00–K92<br>Diseases of the Genitourinary System: N00–N98<br>Certain Conditions Originating in the Perinatal Period: P00–P96<br>Congenital Malformations, Deformations, and Chromosomal Abnormalities: Q00–Q99<br>Symptoms, Signs, and Abnormal Clinical and Laboratory Findings, Not Elsewhere Classified: R00–R99<br>External Causes of Morbidity and Mortality: V01–Y89
Ischemic stroke	I63.0, I63.1, I63.2, I63.3, I63.4, I63.5, I63.6, I63.8, I63.9, I64
Hospitalization for heart failure	I50.1, I50.20, I50.21, I50.22, I50.23, I50.30, I50.31, I50.32, I50.33, I50.40, I50.41, I50.42, I50.43, I50.810, I50.811, I50.812, I50.813, I50.814, I50.81, I50.82, I50.83, I50.84, I50.89, I50.9
Composite of ventricular tachycardia (VT)/ ventricular fibrillation (VF)/ cardiac arrests (CA)	VT - I472<br>VF - I490<br>CA - I46

Supplemental Table S2: Comparison of some clustering methods considered for this study

Clustering Method	Advantages	Limitations	Relevance to Study
**Agglomerative Hierarchical Clustering**[1,2]	▪ Handles small to medium datasets effectively.<br>▪ Provides an interpretable depiction of hierarchical relationships via dendrograms – enables visualisation of clusters and subclusters at different levels.<br>▪ The number of clusters does not need to be predefined.<br>▪ Flexibility with distance metrics and linkage criteria.<br>▪ Hierarchical algorithms show more quality.	▪ Computationally intensive if datasets are very large.<br>▪ Some sensitivity to noise and outliers.	▪ Well-suited for patient stratification due to its hierarchical structure.<br>▪ Provides clinically meaningful and visually interpretable clusters.<br>▪ No assumptions about cluster shapes.
**K-Means Clustering**[1–3]	▪ Simple and easy to implement.	▪ Number of clusters need to be predefined.	▪ Not suitable for exploratory studies such as this due to its

	▪ Computationally efficient and scalable.<br>▪ Works well for larger datasets with spherical clusters.	▪ Assumes spherical, evenly sized clusters, which may not align with clinical data.<br>▪ More sensitive to noise and outliers.	assumption of even cluster shapes and need to predefine the cluster count which may result in loss of information.
**Gaussian Mixture Models (GMM)**[3–6]	▪ Can handle more complex and mixed data<br>▪ Captures overlapping clusters.<br>▪ Accounts for variance and provides probabilistic cluster assignments.<br>▪ Able to handle outliers	▪ Assumes Gaussian distribution, which may not be the case always.<br>▪ Results are sensitive to initialization.<br>▪ Computationally intensive.	▪ Data not normally distributed<br>▪ Can be difficult to interpret results<br>▪ Computationally expensive and prone to numerical instability.

Supplemental Table S3 - STROBE Statement—checklist of items that should be included in reports of observational studies.

STROBE Statement—checklist of items that should be included in reports of observational studies

	Item No.	Recommendation	Page No.	Relevant text from manuscript
**Title and abstract**	1	(*a*) Indicate the study's design with a commonly used term in the title or the abstract	3	Included in the abstract
		(*b*) Provide in the abstract an informative and balanced summary of what was done and what was found	3	Structured abstract
**Introduction**				
Background/rationale	2	Explain the scientific background and rationale for the investigation being reported	5-6	Introduction section
Objectives	3	State specific objectives, including any prespecified hypotheses	5-6	Introduction section
**Methods**				
Study design	4	Present key elements of study design early in the paper	6-9	Methods section under subheading study design and population
Setting	5	Describe the setting, locations, and relevant dates, including periods of recruitment, exposure, follow-up, and data collection	6-9	Methods section under subheading study design and population
Participants	6	(*a*) *Cohort study*—Give the eligibility criteria, and the sources and methods of selection of participants. Describe methods of follow-up<br>*Case-control study*—Give the eligibility criteria, and the sources and methods of case ascertainment and control selection. Give the rationale for the choice of cases and controls<br>*Cross-sectional study*—Give the eligibility criteria, and the sources and methods of selection of participants	6-9	Methods section under subheadings 'Study design and population' and 'Follow-up and outcomes'.
		(*b*)*Cohort study*—For matched studies, give matching criteria and number of exposed and unexposed<br>*Case-control study*—For matched studies, give matching criteria and the number of controls per case	NA	NA

Variables	7	Clearly define all outcomes, exposures, predictors, potential confounders, and effect modifiers. Give diagnostic criteria, if applicable	6-9	Methods section<br>Also included in the Supplemental data (Supplemental Table 1)
Data sources/ measurement	8*	For each variable of interest, give sources of data and details of methods of assessment (measurement). Describe comparability of assessment methods if there is more than one group	6-9	Methods section under subheadings 'Study design and population' and 'Statistical analysis'.
Bias	9	Describe any efforts to address potential sources of bias	6-9	Methods section
Study size	10	Explain how the study size was arrived at		Methods section under subheadings 'Study design and population'

Continued on next page

Quantitative variables	11	Explain how quantitative variables were handled in the analyses. If applicable, describe which groupings were chosen and why	6-9	Methods section under subheading 'Statistical analysis'.
Statistical methods	12	(*a*) Describe all statistical methods, including those used to control for confounding	6-9	Methods section under subheading 'Statistical analysis'.
		(*b*) Describe any methods used to examine subgroups and interactions	6-9	Methods section under subheading 'Statistical analysis'.
		(*c*) Explain how missing data were addressed	6-9	Methods section under subheading 'Statistical analysis'.
		(*d*) *Cohort study*—If applicable, explain how loss to follow-up was addressed<br>*Case-control study*—If applicable, explain how matching of cases and controls was addressed<br>*Cross-sectional study*—If applicable, describe analytical methods taking account of sampling strategy	N/A	No loss to follow-up
		(*e*) Describe any sensitivity analyses	N/A	Not performed.
**Results**				
Participants	13*	(a) Report numbers of individuals at each stage of study—eg numbers potentially eligible, examined for eligibility, confirmed eligible, included in the study, completing follow-up, and analysed	9-11	Results section
		(b) Give reasons for non-participation at each stage	N/A	N/A
		(c) Consider use of a flow diagram	N/A	N/A
Descriptive data	14*	(a) Give characteristics of study participants (eg demographic, clinical, social) and information on exposures and potential confounders	9-11	Results section, under subheading 'Baseline characteristics'
		(b) Indicate number of participants with missing data for each variable of interest	N/A	N/A
		(c) *Cohort study*—Summarise follow-up time (eg, average and total amount)	11	Results section, under subheading 'Major adverse cardiovascular events'.
Outcome data	15*	*Cohort study*—Report numbers of outcome events or summary measures over time	11-13	Results section
		Case-control study—Report numbers in each exposure category, or summary measures of exposure	N/A	N/A
		Cross-sectional study—Report numbers of outcome events or summary measures	N/A	N/A

Main results	16	(*a*) Give unadjusted estimates and, if applicable, confounder-adjusted estimates and their precision (eg, 95% confidence interval). Make clear which confounders were adjusted for and why they were included	11-13	Results section
		(*b*) Report category boundaries when continuous variables were categorized	N/A	N/A
		(*c*) If relevant, consider translating estimates of relative risk into absolute risk for a meaningful time period	N/A	N/A

Continued on next page

Other analyses	17	Report other analyses done—eg analyses of subgroups and interactions, and sensitivity analyses	N/A	N/A
**Discussion**				
Key results	18	Summarise key results with reference to study objectives	13	Discussion section (main body)
Limitations	19	Discuss limitations of the study, taking into account sources of potential bias or imprecision. Discuss both direction and magnitude of any potential bias	17-18	Discussion section under subheading 'Limitations'
Interpretation	20	Give a cautious overall interpretation of results considering objectives, limitations, multiplicity of analyses, results from similar studies, and other relevant evidence	13-18	Discussion section (main body)
Generalisability	21	Discuss the generalisability (external validity) of the study results	16-17	Discussion section including 'Limitations'
**Other information**				
Funding	22	Give the source of funding and the role of the funders for the present study and, if applicable, for the original study on which the present article is based	28	After the manuscript text under subheading 'funding'

*Give information separately for cases and controls in case-control studies and, if applicable, for exposed and unexposed groups in cohort and cross-sectional studies.
